# Hexagonal Boron Nitride as an Intermediate Layer for Gallium Nitride Epitaxial Growth in Near-Ultraviolet Light-Emitting Diodes

**DOI:** 10.3390/ma16227216

**Published:** 2023-11-17

**Authors:** Ah-Hyun Park, Tae-Hoon Seo

**Affiliations:** 1 R&D Center, Flyer, Daejeon 34141, Republic of Korea; ahpark14@gmail.com; 2 Green Energy & Nano Technology R&D Group, Korea Institute of Industrial Technology, Gwangju 61012, Republic of Korea

**Keywords:** hexagonal boron nitride, GaN, graphene, near-ultraviolet light-emitting diode

## Abstract

We introduce the development of gallium nitride (GaN) layers by employing graphene and hexagonal boron nitride (h-BN) as intermediary substrates. This study demonstrated the successful growth of GaN with a uniformly smooth surface morphology on h-BN. In order to evaluate the crystallinity of GaN grown on h-BN, a comparison was conducted with GaN grown on a sapphire substrate. Photoluminescence spectroscopy and X-ray diffraction confirmed that the crystallinity of GaN deposited on h-BN was inferior to that of GaN grown on conventional GaN. To validate the practical applicability of the GaN layer grown on h-BN, we subsequently grew an NUV-LED structure and fabricated a device that operated well in optoelectrical performance experiments. Our findings validate the potential usefulness of h-BN to be a substrate in the direct growth of a GaN layer.

## 1. Introduction

The epitaxial development of GaN-based light-emitting diodes (LEDs) has brought about a significant transformation in the solid-state lighting sector [1,2,3,4]. GaN-based near-ultraviolet LEDs (NUV-LEDs) emitting light in the 370–400 nm range are employed as effective excitation sources for inorganic and organic luminescent materials used in white light production [5,6] and have been extensively used in various applications over the past few decades due to significant advancements in device efficiency, durability, and stability within the field of technology [7,8]. While the latter two qualities are, to a large extent, inherent to the material’s properties, the device efficiency, i.e., the sum of internal and external quantum efficiencies, is principally decided by the structural and optical quality of the active layers and the device configuration. To optimize the internal quantum efficiency to its maximum extent, the most ideal approach is to grow the active layers on a native GaN substrate. GaN-based NUV-LEDs that are constructed via hetero-epitaxial growth on c-plane sapphire, SiC, or Si, as native GaN substrates, are prohibitively expensive. Among several substrates, the c-plane sapphire has many merits, including high-temperature resistance, preservation of the hexagonal crystal structure, and cost-effectiveness. However, the direct growth of an NUV-LED on a sapphire substrate leads to a low-quality layer and the eventual deterioration of device performance because of significant differences in the fundamental properties of the GaN layer and the sapphire substrate, such as lattice constants and thermal expansion coefficients, resulting in highly dense threading dislocations (TDs) [9,10]. It is noteworthy that this mismatch problem is a primary hurdle that needs to be addressed to achieve highly efficient NUV-LEDs.

Recently, the epitaxial growth of sp^3^-bonded group-III-nitrides on sp^2^-bonded two-dimensional (2D) materials, such as hexagonal boron nitride (h-BN) or graphene, has garnered significant attention due to its remarkable physical characteristics, which include high thermal conductivities, chemical and thermal stability, and mechanical flexibility [11,12,13,14,15,16,17,18,19,20,21]. In this case, the 2D material serves a dual purpose, functioning both as a buffer layer featuring a hexagonal in-plane lattice arrangement and as a release layer for mechanically exfoliating the layer. This approach has the potential to enable the production of large-scale flexible III-nitride devices. Achieving the direct epitaxial growth of a GaN layer on 2D materials poses challenges, primarily stemming from the low surface energies of 2D materials due to the absence of dangling bonds along the c-plane [11,12,13,14,15,16,17,18,19]. To resolve this issue, several pioneering studies have demonstrated that the epitaxial growth of a GaN layer on graphene or h-BN can be realized by integrating additional processing that forms dangling bonds or by adding layers, such as zinc oxide nano-walls [11,12], AlN [13,15,18], carbon nanotubes [17], and nanorods [20]. In addition, Wu et al. introduced the growth of an AlGaN-based deep-ultraviolet LED structure on oxygen-plasma-treated h-BN/Al_2_O_3_ [22]. Liu et al. also reported the interfacial bonding behavior and nucleation phenomena of a GaN layer grown on an activated h-BN/sapphire substrate [19]. Although the growth of a GaN layer on graphene or h-BN has been reported, differences in the nucleation behavior of a GaN layer grown on graphene and those grown on h-BN have seldom been reported.

In this study, we utilized graphene and h-BN as intermediary substrates to analyze disparities in the nucleation characteristics of GaN layers grown on them. It was successfully deposited on h-BN/sapphire substrates with the aid of a traditional low-temperature GaN buffer layer. Note that although a flat GaN layer has been successfully grown on both graphene and h-BN by pioneering works [11,15,19], growing GaN on graphene is not as easily accessible as that on h-BN. We also grew GaN-based NUV-LEDs based on these planar GaN layers on the h-BN/sapphire substrate and compared their performance with that of conventional sapphire-grown NUV-LEDs.

## 2. Materials and Methods

### 2.1. Synthesis of h-BN

A large-scale h-BN layer investigated in this work was synthesized on 35 µm thick Cu foils (sourced from Nippon Mining) using low-pressure chemical vapor deposition (LPCVD). The copper foil’s surface was made flat via an electrochemical polishing (ECP) process, conducted in a solution containing phosphoric acid and water for 10 min at 1.8 V. In this process, a Cu plate was employed as the cathode, whereas a 100 × 100 mm copper foil served as the working electrode. Borazine (B_3_N_3_H_6_), as a precursor to h-BN, was stored in a bubbler-equipped canister in a chiller at −10 °C. Following this, the copper foil that had been treated with ECP was placed in the middle position of a quartz tube and heated using a split-tube furnace. Meanwhile, the quartz tube was pumped down to 0.018 torr, and H_2_ gas was flown through the reactor at 15 sccm during the temperature ramp-up up to 1040 °C. The annealing step was carried out at 1040 °C for 60 min under flowing H_2_ gas (15 sccm) at a specified low pressure. The h-BN was synthesized by introducing a mixture of borazine (0.3 sccm) and H_2_ (70 sccm) at 1040 °C for 90 s at a pressure of 5 × 10^−3^ torr. These conditions are ideal for achieving high-quality uniform h-BN. The sample was subsequently rapidly cooled to room temperature in a hydrogen environment, after which the h-BN was transferred onto various substrates, including sapphire, glass, and SiO_2_/Si, using a technique similar to that used to transfer graphene, in order to apply various substrates and investigate the structural properties of h-BN. Additional information is provided in our previous report [23].

### 2.2. Synthesis of Graphene

The graphene that was applied in our study was produced on the ECP-treated copper foil by the use of LPCVD. The copper foil was introduced into a quartz tube with a diameter of 4 inches and placed under an ongoing temperature increase of up to 1030 °C for a duration of 60 min. This process was carried out in the environment of H_2_ gas flowing at a rate of 15 sccm using a split-tube furnace. At the same time, the chemical vapor deposition (CVD) chamber conducted a vacuum process, lowering the pressure to 0.072 torr. Following that, copper foils underwent an annealing process for 60 min. To obtain high-quality graphene, we employed the two-step growth method. In the initial stages, the attainment of large-size graphene domains was successfully achieved on a copper foil via the reduction in nucleation sites in an ambient atmosphere consisting of a mixture of CH_4_ (5 sccm) and H_2_ (100 sccm) gases. This process was carried out for a duration of 60 s at a temperature of 1030 °C. In the next step, the continuous graphene surface was accomplished by enlarging the flow rate of CH_4_ (13 sccm) and increasing the growth time to 8 min. This was carried out while keeping the H_2_ flow rate and temperature identical. The chamber was finally cooled to room temperature by injecting Ar gas (500 sccm). Further details are presented in a previous report [24].

### 2.3. 2D Materials (h-BN or Graphene) Transfer

In order to protect and transfer the 2D materials onto the desired substrate, polymethylmethacrylate (PMMA) was placed onto the surface of 2D materials using a spin-coating process. The spin-coating was carried out at a speed of 4000 rpm for 50 s. Prior to the 2D materials transfer, the unintentional formation of 2D materials on the opposite side of the copper foil was eliminated via the utilization of O_2_ plasma etching. Subsequently, the copper foil with PMMA-covered 2D materials was immersed in a solution containing 0.1 M ammonium persulfate [(NH_4_)_2_S_2_O_8_] for 4 h, allowing the process of etching the copper foil. Then, PMMA/2D materials were transferred onto various substrates, such as the 300 nm SiO_2_/Si substrate, the c-plane sapphire, glass, and Cu mesh TEM grid, to investigate the properties of 2D materials and to grow GaN. The PMMA was removed using acetone, and the sample was annealed for 2 h at 500 °C in an Ar/H_2_ gas mixture to eliminate any remaining PMMA residues.

### 2.4. Growth of un-Doped GaN on an h-BN/Sapphire Substrate

A GaN epilayer was formed using metal–organic chemical vapor deposition (MOCVD) on an h-BN/sapphire substrate under identical growth conditions to those used to directly grow the GaN layer on a sapphire substrate. A 25 nm thick GaN buffer layer was applied to a sapphire substrate at 560 °C for 100 s under a growth pressure of 400 mbar as part of the conventional GaN growth process. Following this, a 3.7 µm thick undoped GaN layer was grown for 2 h at 1130 °C and 100 mbar. 

### 2.5. Growth and Fabrication of the NUV-LED Structure

**Figure 1:**
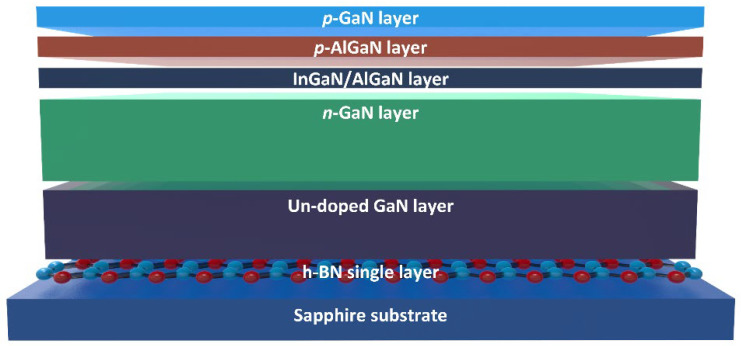
Schematic diagram of InGaN/AlGaN NUV-LED wafer grown on h-BN/sapphire substrate.

The LED configuration included an undoped GaN layer, a Si-doped n-type GaN layer, five sets of InGaN/AlGaN multi-quantum wells (MQWs), a Mg-doped p-AlGaN electron-blocking layer (EBL), and a Mg-doped p-type GaN layer. A 2 µm thick Si-doped n-type GaN layer was deposited on the undoped GaN layer on the h-BN/sapphire substrate at 1100 °C and 400 mbar for 60 min. Following this, five sets of In_0.04_Ga_0.96_N quantum wells and Al_0.08_Ga_0.92_N barrier layers, each with thicknesses of 3 and 12 nanometers, were grown at 720 °C and 810 °C, respectively, to serve as the active layers. Then, a 25 nm thick Mg-doped p-Al_0.25_Ga_0.75_N electron-blocking layer (EBL) and a 100 nm thick p-type GaN contact layer were deposited at 1040 °C. To activate the Mg dopants, rapid thermal annealing was performed in a nitrogen (N_2_) atmosphere at 940 °C for 40 s. The *p*-type GaN layer was calculated to have a hole concentration of approximately 10^16^ cm^−3^. Figure 1 illustrates a schematic representation of an InGaN/AlGaN NUV-LED wafer developed on an h-BN/sapphire substrate. Further details are provided in the report of Seo et al. [25]. After LED wafer growth, individual LEDs, each with a chip dimension of 350 × 350 µm, were manufactured by utilizing an inductively coupled plasma etcher (ICP) with Cl_2_/BCl_3_/Ar gases to delineate the mesa region until the n-type GaN layer was revealed for contact with the n-electrode. Using an electron beam evaporator, a 200 nm thick layer of indium tin oxide (ITO) was then applied as a transparent current-spreading electrode on the *p*-type GaN layer. Finally, 50 nm thick Cr and 250 nm thick Au layers were deposited onto both the *n*-type GaN and ITO layers to serve as the *n*- and *p*-electrodes, employing an electron beam evaporator.

### 2.6. Formation of Patterned 2D Materials

The CVD-grown 2D materials were transferred onto a c-plane sapphire substrate to fabricate patterned 2D materials. Subsequently, the designated area of 3 × 3 µm was coated with a photoresist (PR), acting as a protective mask against the etchant. The 2D materials were then patterned by exposing them to an ICP using O_2_ plasma. Finally, the PR was removed using acetone.

### 2.7. Characterization

Field-emission scanning electron microscopy (SEM) was employed to examine the surface structures of h-BN on copper foil, the initial phase of the GaN buffer layer on the h-BN/sapphire substrate, and the subsequent undoped GaN layer grown on these substrates. X-ray photoelectron spectroscopy (XPS) was conducted with the use of monochromatic aluminum K-alpha X-rays, utilizing equipment from Thermo Fisher Scientific. High-resolution transmission electron microscopy (HRTEM) was performed using an FEI TITAN G2 instrument equipped with an image Cs corrector and a monochromator provided by Thermo Fisher Scientific in Waltham, MA, USA. The equipment was operated at an optimized accelerator voltage of 60 kV to avoid damaging the h-BN. The crystallinities of the GaN layers, both with and without h-BN, were assessed via X-ray diffraction (XRD). For a comparative evaluation, samples were scanned at 2°/min in the 20–80° range. Photoluminescence (PL) spectroscopy, excited with the 325 nm line of a He–Cd laser, was employed to investigate the crystalline quality and residual strains in GaN layers cultivated on both sapphire and h-BN/sapphire substrates. Current–voltage (I-V) plots were constructed using a probe-station system, and electroluminescence (EL) experiments were performed.

## 3. Results and Discussion

**Figure 2:**
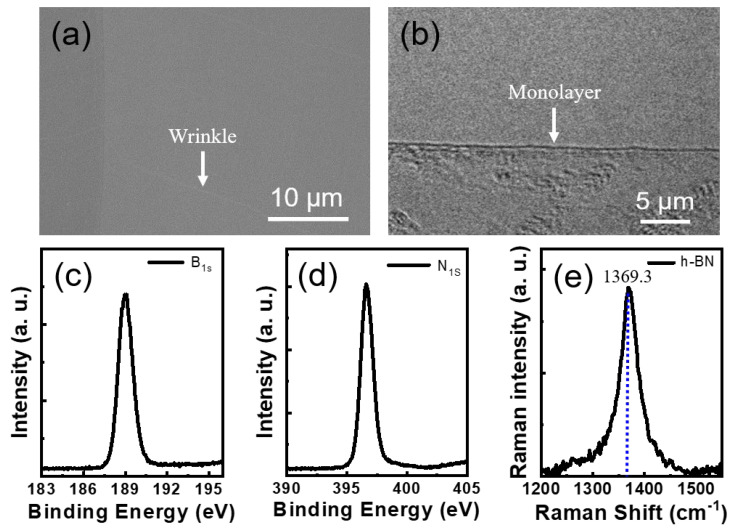
(**a**) SEM image of h-BN grown on copper foil; (**b**) HRTEM image of monolayer h-BN; (**c**,**d**) 1s core-level XPS spectra; and (**e**) Raman spectrum of the h-BN transferred onto the SiO_2_/Si substrate.

In Figure 2a, one can observe an SEM image of h-BN production on copper foil via the ECP process. The h-BN completely covered the copper foil within 90 s of growth and exhibited a seamless two-dimensional nanosheet structure. However, wrinkles are commonly observed as they alleviate thermal stress; they may have originated through defect nucleation on the step margins of copper terraces during quenching, and their presence provides indirect evidence for the successful growth of continuous h-BN [26]. The h-BN thickness was determined using HRTEM, the results of which are depicted in Figure 2b, which verified that the h-BN synthesized in this study is predominantly monolayer. We acquired B and N core-level XPS spectra of the h-BN monolayer on the copper foil to confirm the growth of the h-BM monolayer and to quantify atomic concentrations. Atomic concentrations were determined from the intensity of the core-level photoemission for each element normalized by the atomic sensitivity factor at the photon emission energy. The B-to-N atomic ratio was calculated to be 1.02:0.98, which is close to 1:1. All XPS spectra were Lorentzian-fitted with multiple peaks. Figure 2c,d show B 1s and N 1s peaks at 189.2 and 396.8 eV, respectively, consistent with the binding energies reported for h-BN [27,28]. In addition, Raman spectroscopy was employed, which has become a crucial technique for characterizing and exploring two-dimensional materials to conduct a more in-depth analysis of the crystal structure of the h-BN monolayer. In Figure 2e, the Raman spectrum of the h-BN transferred onto the SiO2/Si substrate exhibits a peak at 1369.3 cm^−1^, which is indicative of the E_2g_ lattice-vibration mode of h-BN in-plane oscillations. The peak at 1370 cm^−1^ corresponds to h-BN, whereas those associated with the cubic structure are observed at 1300 cm^−1^ (for the longitudinal optical vibrational mode) and 1065 cm^−1^ (for the transverse optical vibrational mode). Our sample did not exhibit the vibrational mode of cubic BN. Taking into account the number of h-BN layers, the E_2g_ mode of bulk h-BN is observed at approximately 1366 cm^−1^, whereas the E_2g_ mode of the h-BN single layer is located between 1368 and 1370 cm^−1^, and that of two-to-five-layer thick h-BN is observed between 1364 and 1367 cm^−1^ [29,30]. The identification of a peak at 1369.3 cm^−1^ in the h-BN indicates that it possesses a monolayer structure, which is in accordance with the HRTEM image of the sample presented in Figure 2b. The crystal size can be inferred from the full width at half maximum (FWHM) of the E_2g_ mode due to its correlation with the phonon vibration duration. The prepared h-BN has an FWHM value of approximately 26 cm^−1^, which is comparable to the value of previously reported h-BN [31,32].

**Figure 3:**
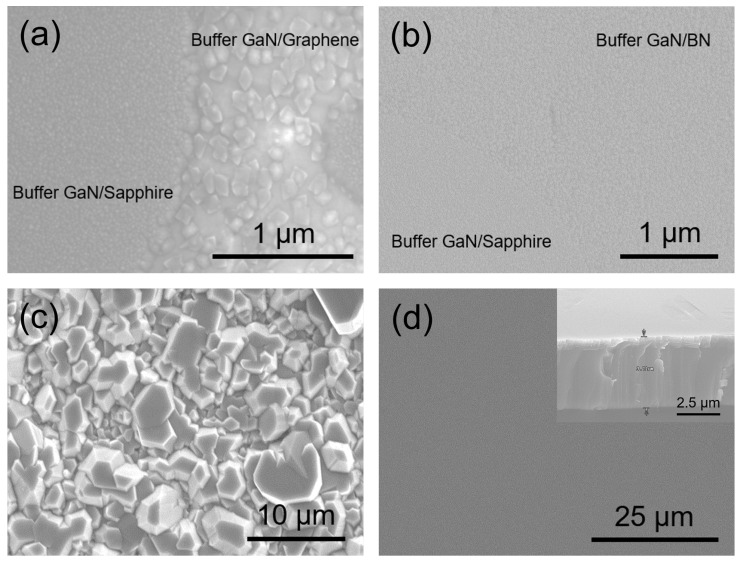
SEM images acquired during the initial step involved in the formation of the GaN buffer layer on (**a**) patterned graphene and (**b**) patterned h-BN. SEM images of undoped GaN formed on (**c**) graphene and (**d**) h-BN. The inset in panel (**d**) displays a cross-sectional SEM image.

The early stages of growth are crucial for achieving epitaxial layers and ensuring the superior quality of the resulting layer. To examine and compare GaN nucleation on h-BN, we acquired SEM images of the GaN buffer layer on a graphene/sapphire substrate and an h-BN/sapphire substrate, each with a 3 × 3 µm pattern size. This experiment enabled the initial GaN growth on sapphire, graphene, and h-BN to be compared. Figure 3a shows that dense and almost homogeneous nucleation occurred as the GaN buffer layer grew on sapphire. However, the GaN buffer layer grown on graphene exhibited irregular and low-density nucleation, which is attributable to the inherent non-reactivity of graphene and stems from its hexagonal arrangement of sp^2^-bonded carbon atoms that lack dangling bonds [11]. The nucleated GaN exhibited an irregular three-dimensional growth pattern, leading to unevenly dispersed nucleation and resulting in a partially covered GaN surface after a growth period of 3 h (Figure 3c). In the case of h-BN (Figure 3b), the GaN buffer layer displays a consistent and organized GaN nucleation morphology, in contrast to the GaN buffer layer on graphene. However, GaN was less densely nucleated on h-BN than the GaN buffer layer grown on sapphire. The B-N bond types provide a reasonable explanation for the differences observed between graphene and h-BN. While the C-C- bonds in graphene are purely covalent and have evenly distributed electrons, the B-N bonds in the h-BN sheets display an alternating ionic/covalent nature due to the significant difference in the electronegativities of B and N [33,34]. We believe that electrically active N atoms attract the gallium atoms, which assists nucleation. First, growth begins only from GaN nucleation seeds that originate from N atoms or imperfections, such as point defects, wrinkles, and folds. These islands increase in size and spread progressively across the h-BN as growth progresses. The GaN crystallites eventually coalesce laterally to cover the entire surface. The GaN buffer layers do not cover the entire surface during the early growth following GaN deposition at low growth temperatures. This activity led to a noticeable transformation of the GaN surface, from a three-dimensional to a two-dimensional structure, on the h-BN during subsequent high-temperature growth, as shown by the SEM image in Figure 2d. The ultimate thickness of the GaN layer was ascertained to be 3.7 µm.

**Figure 4:**
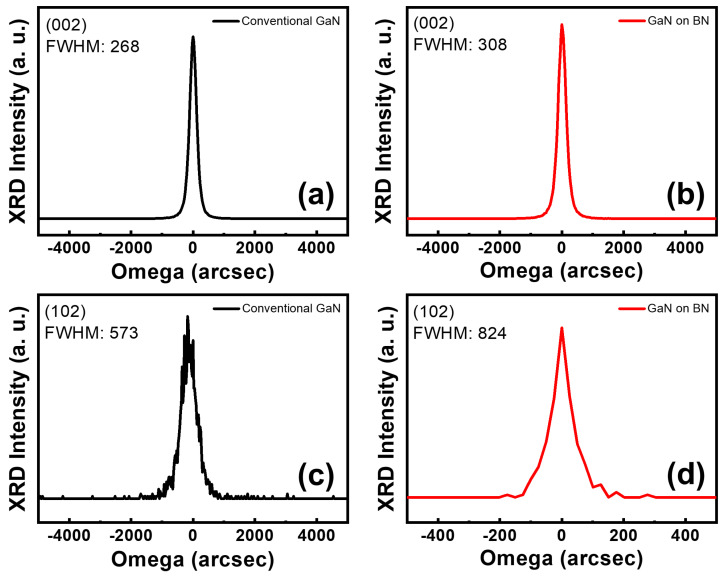
XRD omega rocking curves for the (**a**,**b**) symmetrical (002) and (**c**,**d**) asymmetrical (102) reflections of a GaN epilayer grown on sapphire and h-BN.

We synthesized GaN on h-BN with a crack-free, mirror-like, and flat morphology. However, directly comparing a GaN layer on h-BN and its counterpart on graphene may not provide an entirely accurate result, considering that the GaN layer on graphene does not exhibit a typical two-dimensional morphology. We examined two samples to assess the crystallinity of the GaN layer on h-BN: GaN grown on h-BN and conventional GaN grown on a sapphire substrate. XRD omega-scanning is typically used to evaluate the crystallinity of a GaN layer. Figure 4 shows the XRD rocking curves for both the symmetric (002) and asymmetric (102) planes of GaN on sapphire and h-BN, which are commonly used to identify dislocation types. Specific lattice distortions are well known to influence the omega-scan FWHM values of crystal planes, such that the (002) plane is susceptible to screw or mixed dislocations, whereas the (102) plane is responsive to all types of dislocations, including pure-edge, screw, and mixed [17,35,36]. When compared to a conventional GaN layer grown on sapphire, the GaN grown on h-BN exhibits a slightly larger FWHM value for its (002) plane (308 vs. 268 arcsec), whereas the FWHM of the (102) plane is significantly larger (824 vs. 573 arcsec). Despite the slightly higher FWHM value for the (002) plane, the considerably higher value for the (102) plane implies the existence of more pure-edge dislocations in the GaN in h-BN than those on sapphire. The results presented herein suggest that the crystallinity of the GaN grown on h-BN is inferior to that grown on sapphire.

**Figure 5:**
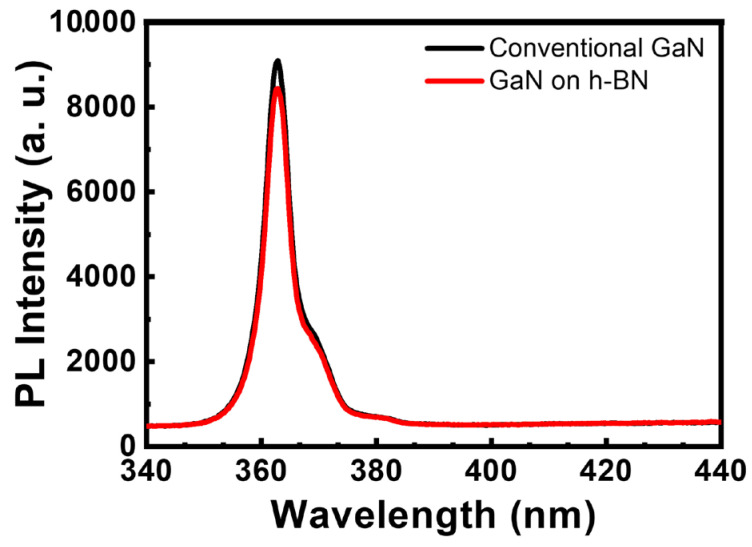
Room-temperature PL spectra of GaN epilayer formed on sapphire and h-BN.

The impact of h-BN on the optical characteristics of the GaN layer was examined using PL spectroscopy, a rapid and nondestructive method, the results of which are depicted in Figure 5. The PL spectrum is notably influenced by the strain state and epilayer defects. Figure 5 depicts the room-temperature photoluminescence (PL) spectra of GaN grown separately on h-BN and on a sapphire substrate. Similar strong near-band-edge (NBE) emission peaks were observed at approximately 362 nm for both samples; however, GaN grown on h-BN exhibited an NBE emission peak intensity that was approximately 8% less intense than that grown on sapphire. The intensity of the NBE emission peak is generally acknowledged to be closely related to the defect density [37]. Although we successfully grew a smooth and crack-free GaN layer on h-BN, the GaN layer formed on h-BN appeared to contain additional defects, which is in good accord with the XRD results shown in Figure 4.

**Figure 6:**
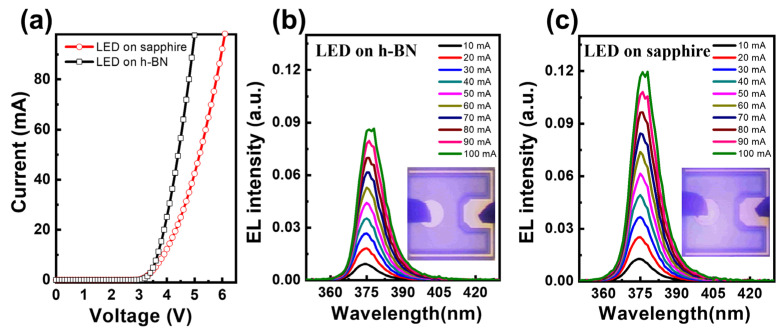
(**a**) I-V curves and (**b**,**c**) EL intensities with an injection current of fabricated NUV-LEDs on h-BN and sapphire. The insets display EL images at an applied current of 20 mA.

We used a two-dimensional GaN layer on h-BN to fabricate NUV-LEDs, and LED devices with and without h-BN were fabricated to understand the real potential of the GaN layer grown on h-BN. We examined the effect of h-BN on the optoelectrical performance of the constructed NUV-LEDs. Figure 6a compares the I-V characteristics of an NUV-LED fabricated on h-BN with that fabricated on a sapphire substrate. The NUV-LEDs exhibited forward voltages of 3.25 V (on sapphire) and 3.37 V (on h-BN) at a driving current of 20 mA. The forward voltage of the NUV-LED on h-BN is slightly elevated compared to that on sapphire, which is rationalized by the higher number of defects and the greater internal strain in the GaN layer grown on h-BN. In general, TDs in LEDs serve as pathways for current leakage, thereby possibly increasing the forward voltage [38,39]; consequently, they serve as non-radiative recombination centers. The EL spectra of the NUV-LEDs fabricated on h-BN and sapphire substrates are shown in Figure 6b,c, respectively, which were obtained by varying the injection current in the 10–100 mA range. Both samples demonstrated adequate stability when tested at injection currents of up to 100 mA. The EL emission wavelength was observed to slightly redshift with increasing applied current (from 375 nm at 10 mA to 378 nm at 100 mA). The blue InGaN/GaN LED exhibited a lower emission peak energy than the theoretical value due to the quantum confinement Stark effect (QCSE). This shift originates from strong spontaneous polarization and internal piezoelectric fields associated with the high indium mole fraction. The peak position of the blue LED underwent a progressive shift toward higher energies with increasing excitation power, which is attributable to a higher photogenerated-carrier density leading to a lower QCSE and resulting in a blueshift in the emission peak as a consequence. The redshift behavior of the NUV-LEDs is a result of the relatively weak QCSE induced by a lower indium mole fraction [40]. The EL emission intensity of the NUV-LED on h-BN is 30% lower than that of the NUV-LED on the sapphire substrate at an injection current of 20 mA. The insets in Figure 6b,c present the electroluminescence (EL) emission images of the constructed devices at an injection current of 20 mA. The NUV-LED on the h-BN substrate is less bright than that on the sapphire substrate. Commercially advancing h-BN as a GaN growth substrate requires achieving a level of quality that exceeds that of the GaN layer grown on sapphire. We believe that a high-quality GaN layer on h-BN can be achieved via material optimization using active learning and lateral epitaxial overgrowth methods. Nevertheless, the findings of this study validate the potential utility of h-BN as a substrate.

## 4. Conclusions

We employed graphene and h-BN as intermediate substrates to conduct a comparative analysis of the nucleation characteristics of GaN grown on these substrates. Although attempts to grow GaN on graphene failed to achieve a two-dimensional morphology, we successfully grew GaN on h-BN. The crystallinity of the GaN grown on h-BN was established via XRD and PL spectroscopy, with the results compared to those obtained using conventional GaN grown on sapphire. These findings reveal that the GaN produced on h-BN was less crystalline than that grown on sapphire despite the successful growth of crack-free GaN on h-BN. Planar GaN grown on h-BN forms a building block for the subsequent growth of a GaN-based NUV-LED; consequently, we fabricated LED devices. The optoelectrical performance of the manufactured NUV-LEDs was investigated to assess the effect of h-BN, with the LED grown on h-BN demonstrating excellent operational properties. This study demonstrated the potential applicability of h-BN as a substrate for GaN growth. We believe that the use of various growth strategies may enable h-BN substrates to surpass conventional sapphire substrates in terms of GaN growth quality.

## Data Availability

Data supporting the findings of this study is available from the corresponding author upon request.
